# Spatial Distribution and Phenotypic Profiling of Cd68^+^ and Cd163^+^ Macrophages in Melanoma Progression: Insights into Tumor Microenvironment Dynamics

**DOI:** 10.3390/biomedicines13092178

**Published:** 2025-09-05

**Authors:** Grigory Demyashkin, Dmitrii Atiakshin, Kirill Silakov, Vladimir Shchekin, Maxim Bobrov, Matvey Vadyukhin, Tatyana Borovaya, Ekaterina Blinova, Petr Shegay, Andrei Kaprin

**Affiliations:** 1 Department of Digital Oncomorphology, National Medical Research Centre of Radiology, 125284 Moscow, Russia; 2 Laboratory of Histology and Immunohistochemistry, Institute of Translational Medicine and Biotechnology, I.M. Sechenov First Moscow State Medical University (Sechenov University), 119048 Moscow, Russia; 3 Research and Educational Resource Center for Immunophenotyping, Digital Spatial Profiling and Ultrastructural Analysis Innovative Technologies, Peoples’ Friendship University of Russia (RUDN University), 117198 Moscow, Russia; 4 Department of Fundamental Medicine, Institute for Physics and Engineering in Biomedicine, MEPhI, National Research Nuclear University, 115409 Moscow, Russia; 5 Department of Urology and Operative Nephrology, Peoples’ Friendship University of Russia (RUDN University), 117198 Moscow, Russia

**Keywords:** melanoma, macrophages, TAMs, M1 and M2 phenotypes, CD68, CD163

## Abstract

**Background**: Macrophages are key components of the tumor microenvironment and play a critical role in melanoma progression. However, the dynamics of different macrophage subsets, particularly CD68^+^ and CD163^+^ populations, in relation to tumor thickness and stage remain insufficiently characterized. **Objective:** This study aimed to quantitatively assess intratumoral and peritumoral CD68^+^ and CD163^+^ macrophages in cutaneous melanoma and to investigate their associations with Breslow thickness, pT stage, and AJCC stage. **Methods**: We conducted a retrospective cohort study of 126 patients with cutaneous melanoma (AJCC stages IA–IIID). Tumor samples were examined histologically and immunohistochemically for CD68^+^ and CD163^+^ macrophages, and quantitative analysis was performed in intratumoral and peritumoral regions. **Results**: This study included 126 patients with cutaneous melanoma, ranging in stage from IA to IIID. Histopathological analysis revealed that melanoma tissues were primarily composed of irregular clusters of atypical melanocytic cells infiltrating the dermis and subcutaneous fat. Immunohistochemical staining identified CD68^+^ and CD163^+^ macrophages both within the tumor and in the surrounding stroma. Macrophage infiltration significantly increased with tumor thickness (Breslow) and progression to more advanced stages. Specifically, at Breslow thickness <1.0 mm, the mean number of CD68^+^ macrophages in the intratumoral zone was 29.7 ± 4.3 cells, increasing to 70.3 ± 6.4 cells in tumors >4.0 mm. CD163^+^ macrophages showed similar trends, with a rise from 15.6 ± 2.8 cells at <1.0 mm to 39.8 ± 4.6 cells at >4.0 mm in the intratumoral zone. Additionally, macrophage density was higher in tumors with ulceration, and both CD68^+^ and CD163^+^ macrophage numbers increased progressively with tumor stage, particularly in advanced stages. The number of CD68^+^ macrophages at stage IA in the intratumoral zone was 21.6 ± 3.1 cells and increased to 56.4 ± 6.8 cells at stage IIID, while CD163^+^ macrophages rose from 13.8 ± 3.2 cells at stage IA to 36.4 ± 4.6 cells at stage IIID. This suggests that macrophage infiltration, particularly CD163^+^ cells, correlates with melanoma progression. **Conclusions**: Our findings highlight distinct spatial and phenotypic patterns of macrophage infiltration in melanoma progression. The prominent increase in CD68^+^ and CD163^+^macrophages suggests their important role in tumor-associated immunomodulation. Further studies are warranted to elucidate macrophage polarization states and their prognostic and therapeutic implications in melanoma.

## 1. Introduction

Melanoma is one of the most aggressive types of malignant skin tumors, accounting for approximately 20% of all cases [1]. Over the past 50 years, its incidence has increased significantly, primarily among fair-skinned populations, especially in developed countries where ultraviolet (UV) radiation is the main risk factor [2]. The highest prevalence is observed in North America, Europe, and Australia, whereas in Asia, Africa, and Latin America, melanoma is less common but associated with higher mortality rates due to delayed diagnosis [3]. In recent decades, melanoma-related mortality has been declining, largely due to improvements in treatment strategies and earlier detection [4].

Cutaneous melanoma is considered an immunogenic tumor, which is histologically characterized by a dense peritumoral infiltrate that, together with the surrounding stroma, constitutes the tumor microenvironment (TME) [5]. The cellular composition of the TME is heterogeneous and includes fibroblasts, immune cells, adipocytes, vascular endothelial cells, and others. These cells interact with atypical melanocytes, with each other, and with components of the extracellular matrix through secreted proteins (such as matrix metalloproteinases) and growth factors (such as transforming growth factor-β, epidermal growth factor, etc.) [6,7,8]. Among these cellular players, tumor-associated macrophages are of particular interest due to their prominent role in melanoma tumor development and progression.

Cutaneous melanoma comprises several histological subtypes that differ in frequency, clinical presentation, and molecular background. The most common variant is superficial spreading melanoma (SSM), reported in approximately 66–78% of cases, followed by nodular melanoma (NM), which accounts for about 14–15%, and acral lentiginous melanoma (ALM), observed in 15–20% of patients in certain populations, particularly in Latin America and Asia [9,10]. In addition, uncommon variants collectively account for less than 10% of melanomas, including desmoplastic, spitzoid, nevoid, amelanotic, and other rare subtypes, which often present diagnostic challenges due to their atypical morphology and clinical features [11]. These subtypes are also characterized by distinct genetic alterations that drive tumorigenesis. Mutations of the *BRAF* gene are the most frequent, detected in approximately 38.5% of melanomas, while *NRAS* mutations occur in about 16.4% and *KIT* mutations in around 10% of cases. *BRAF* mutations are most commonly associated with superficial spreading melanoma and tumors located on the trunk, whereas *NRAS* mutations correlate with nodular melanoma and extremity localization. *KIT* mutations, although less frequent, are typically observed in acral and mucosal melanomas [12]. The identification of these driver mutations has important clinical relevance, as they contribute to tumor initiation and progression and serve as potential therapeutic targets in precision oncology.

Macrophages within the tumor microenvironment of cutaneous melanoma comprise two major subpopulations with opposing functions. Immunohistochemical analysis using CD68 and CD163 markers enables the identification of pro-inflammatory M1 macrophages (CD68^+^/pSTAT1^+^) and immunosuppressive M2 macrophages (CD68^+^/CD163^+^) [13]. Studies of the melanoma tumor microenvironment consistently demonstrate a marked predominance of the M2 phenotype, whereas M1-positive cells are relatively scarce. Notably, a high density of M2 macrophages correlates significantly with poor prognosis, underscoring the critical role of the balance between M1 and M2 macrophage subpopulations in the progression of cutaneous melanoma [14].

Despite significant progress in understanding the role of macrophages in cutaneous melanoma, the quantitative characteristics of CD68^+^ and CD163^+^ cells at different stages of the disease remain insufficiently studied. In particular, the dynamics of macrophage infiltration across stages I–III, in the absence of distant metastases, require further clarification. This study aims to address this gap by providing a comprehensive analysis of CD68^+^ and CD163^+^ macrophages in cutaneous melanoma stages I–III, which may be critical for elucidating the mechanisms of tumor progression.

## 2. Material and Methods of Research

### 2.1. Study Design and Setting

This retrospective, multicenter cohort study was observational and analytical in design and was conducted spanning 2023–2024 years at National Medical Research Radiological Center (Moscow, Russia). Potential patient cases were initially identified through a search of the electronic medical record systems at both institutions using International Classification of Diseases, 10th Revision (ICD-10) codes corresponding to the primary diagnosis C43—Malignant melanoma of skin.

### 2.2. Participant Selection

The study included data from 126 patients with a confirmed diagnosis of melanoma. Medical records, primarily case histories, were used for analysis. For all patients, anamnesis, clinical manifestations, disease stage according to the American Joint Committee on Cancer (AJCC 8th edition) classification, the nature of surgical and conservative treatments, as well as disease outcomes, were taken into account. Surgical specimens (paraffin blocks) stained with hematoxylin and eosin were also examined, and histological subtypes of melanoma were studied.

Based on the collected data, patient groups were formed corresponding to stages IA, IB, IIA, IIB, IIC, IIIA, IIIB, IIIC, and IIID according to the AJCC 8th edition classification.

Patients were eligible for the study if they had histologically confirmed cutaneous melanoma of AJCC stages IA–IIID without distant metastases (M0). Eligible histological subtypes included superficial spreading melanoma (ICD-O: 8743/3), nodular melanoma (ICD-O: 8721/3), and desmoplastic melanoma (ICD-O: 8745/3). Only patients with preserved functional status (ECOG 0–1) were included.

Exclusion criteria comprised Spitz-type lesions; prior systemic or local antitumor therapy; evidence of distant metastases or recurrent disease; synchronous or metachronous multiple primary cancers; active infectious diseases; and autoimmune disorders.

### 2.3. Histologic Research

Serial 2 μm thick sections were obtained from archival paraffin-embedded tissue blocks of melanoma. Sections were stained with hematoxylin and eosin (H&E). Histological analysis was performed using a Leica DM2000 light microscope with microphotography capabilities (Leica Microsystems, Wetzlar, Germany), in accordance with standard histopathological criteria.

### 2.4. Immunohistochemical (IHC) Assay

Immunohistochemistry was performed according to standard protocols. Primary antibodies used included anti-CD68 (Abcam, Cambridge Biomedical Campus, Cambridge, UK; ab303565, dilution 1:1000) and anti-CD163 (Abcam, Cambridge Biomedical Campus, Cambridge, UK; ab182422, dilution 1:500). For secondary antibody detection, the HiDef Detec-tion™ HRP Polymer System (Cell Marque, Rocklin, CA, USA) was applied. This two-component system included anti-mouse/rabbit IgG antibodies, horseradish peroxidase (HRP), and DAB chromogen substrate. Cell nuclei were counterstained with Mayer’s hematoxylin. Positive controls: Spleen tissue—rich in macrophages—was used for CD68, while tonsil tissue, known for strong macrophage staining, served as the positive control for CD163. Negative controls: Serial sections of tumor were stained using isotype-matched control antibody.

### 2.5. Morphometric Study

The quantification of CD68^+^ and CD163^+^ macrophages was performed by counting tumor buds in hotspot areas. The assessment was carried out on immunohistochemically stained tissue sections. Initially, areas with the highest macrophage density were identified by scanning the slides at low magnification (×40–100) using QuPath version 0.5.1 (Belfast, Northern Ireland, UK) [15]. Depending on the size of the lesion, between three and five hotspot areas were selected for counting: three in cases of small lesions and five for larger tumor areas. Macrophages were counted in both intratumoral regions and the peritumoral stroma. Images were acquired at ×400 magnification using QuPath V0.5.1, and the number of CD68^+^ and CD163^+^ cells in each hotspot was recorded. The final macrophage count for each sample was calculated as the average across all selected hotspots. Macrophages located near necrotic areas were excluded from the analysis. In cases of melanin deposition, CD68^+^ and CD163^+^ macrophages were evaluated based on their cellular and nuclear morphology. For subsequent analyses, tumor thickness was stratified according to the Breslow scale using the cut-off points defined in the AJCC 8th edition (<0.8–1.0 mm, >1.0–2.0 mm, >2.0–4.0 mm, and >4.0 mm), which are clinically relevant for prognosis and widely applied in melanoma studies.

### 2.6. Data Collection

Data for this study were systematically extracted from the electronic medical records system. The collected data were then entered into a secure, encrypted Microsoft Excel spreadsheet (Microsoft Excel 2010; Microsoft Corporation, Seattle, WA, USA) for further processing and analysis. The spreadsheet was subsequently reviewed and verified by several co-investigators from the research team, who performed thorough checks to ensure the accuracy and completeness of the dataset before analysis. This multi-step verification process helped minimize errors and ensured the reliability of the data.

### 2.7. Statistical Analysis

Statistical analysis was performed using SPSS software, version 12.0 (IBM Analytics, Armonk, New York, NY, USA). The Shapiro–Wilk test was used to assess the normality of data distribution. A *p*-value > 0.05 was considered indicative of a normal distribution. Since the data did not follow a normal distribution, intergroup comparisons of quantitative variables were conducted using the non-parametric Mann–Whitney U test. Data in tables are presented as median and interquartile range (Me [Q1–Q3]). A *p*-value < 0.05 was considered statistically significant. Data visualization in the form of violin plots was carried out using in BioRender.com. 

## 3. Results

### 3.1. Characteristics of Patients

Patient characteristics and clinicopathological data are presented in Table 1. The study included 126 patients with a mean age of 64.2 ± 6.8 years. The predominant histo-logical type was nodular melanoma, and the most common tumor localization was the lower extremities (47 cases). AJCC staging ranged from stage IA to IIID, with the highest number of patients classified as stages IIIA–IIID.

### 3.2. Histological Patterns

**Figure 1:**
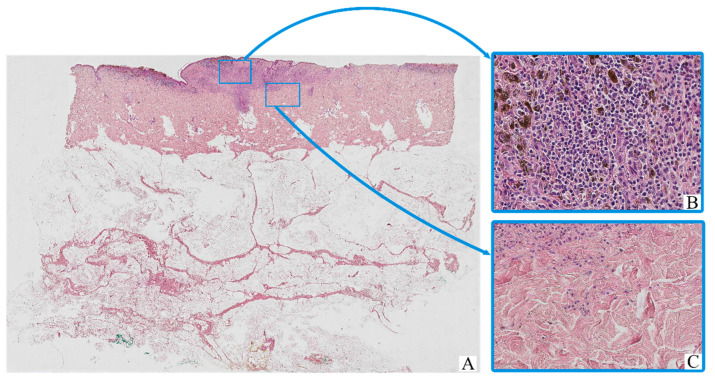
Patient A, female, 61 years. Invasive melanoma, 8743/3—superficial spreading subtype, Breslow thickness 0.9 mm (pT1b). (**A**)—histoscan; (**B**)—tumor, magnification ×200; (**C**)—peritumoral tissue, magnification ×200. Staining: hematoxylin and eosin.

All tumor samples from the study cohort (*n* = 126; 100%) demonstrated histopathological features consistent with cutaneous melanoma (Figure 1). The neoplastic tissue was predominantly composed of irregular nests, sheets, or fascicles of atypical melanocytic cells infiltrating the dermis, often with extension into the subcutaneous fat. The tumor architecture was variably nodular or diffuse, depending on the histological subtype.

The majority of tumor cells exhibited marked cytological pleomorphism. Cells displayed oval, polygonal, or spindle-shaped morphology, with scant to moderate eosinophilic cytoplasm. In many cases, the cytoplasm contained coarse brown pigment granules consistent with melanin. The nuclei were enlarged, hyperchromatic, and often irregular in contour, with prominent nucleoli noted in a substantial proportion of tumor cells.

The tumor stroma was variably fibrotic and frequently showed moderate peritumoral and intratumoral inflammatory infiltrates composed of lymphocytes, plasma cells, and scattered histiocytes. Occasional eosinophils and neutrophils were also present. Areas of intercellular edema, vascular congestion, and focal coagulative necrosis were identified in some samples, particularly in larger or more deeply invasive tumors.

Ulceration of the overlying epidermis was observed in a subset of cases, often accompanied by reactive epidermal hyperplasia at the periphery. Mitotic figures, including atypical mitoses, were readily identified in most tumors, with a mitotic index varying by stage and histological subtype.

Histological staging according to AJCC 8th edition ranged from early superficial involvement (stage IA) to deeply invasive lesions with satellitosis or in-transit metastases (stage IIID). In lower-stage tumors (e.g., stage IA–IIB), the neoplastic process was largely confined to the dermis, whereas advanced-stage tumors (e.g., stage IIIC–IIID) demonstrated extensive infiltration into the subcutis, vascular or lymphatic invasion, and in some cases, involvement of adjacent structures.

### 3.3. Immunohistochemical Assay

Immunohistochemical staining revealed the presence of CD68^+^ and CD163^+^ macrophages in both intratumoral and peritumoral compartments of cutaneous melanoma samples. These cells were predominantly observed as scattered single elements or small clusters, with a tendency to accumulate in areas of dense tumor cell infiltration and stromal remodeling.

**Figure 2:**
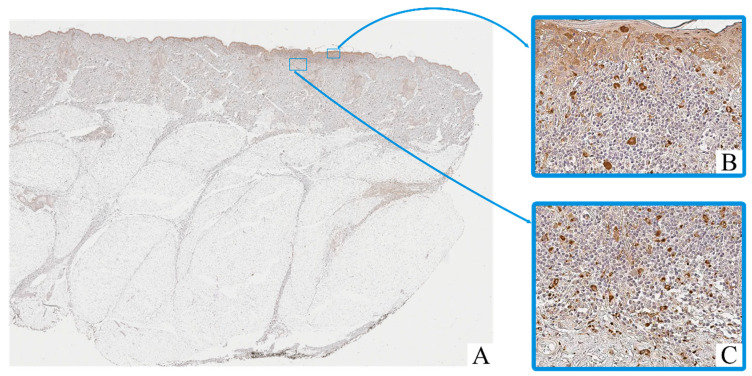
Patient O, male, 52 years. Invasive melanoma, 8743/3 superficial spreading subtype, Breslow thickness 0.4 mm (pT1a). (**A**)—histoscan; (**B**)—intratumoral tissue, magnification ×400; (**C**)—peritumoral tissue, magnification ×400. Immunohistochemical reaction with antibodies to CD68.

**Figure 3:**
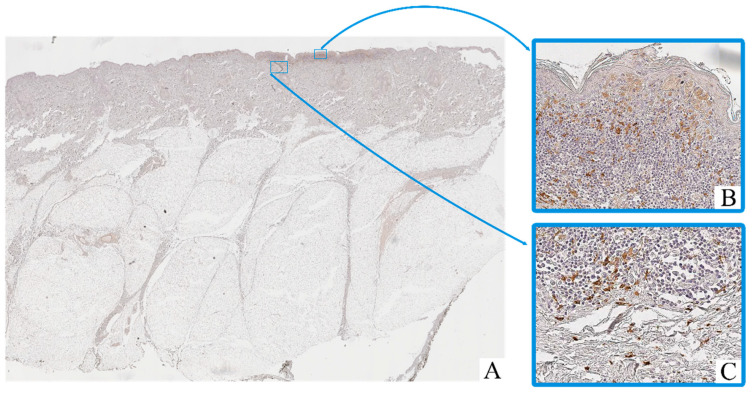
Patient O, male, 54 years. Invasive melanoma, 8743/3 superficial spreading subtype, Breslow thickness 0.4 mm (pT1a). (**A**)—histoscan; (**B**)—intratumoral tissue, magnification ×400; (**C**)–peritumoral tissue, magnification ×400. Immunohistochemical reaction with antibodies to CD163.

CD68^+^ macrophages exhibited moderate to strong cytoplasmic immunoreactivity, often with irregular contours (Figure 2). Their nuclei were oval to polygonal, with variable chromatin patterns and occasional nucleoli. CD163^+^ macrophages displayed a more rounded or spindle-shaped morphology, with distinct membrane-associated staining and smaller, more uniform nuclei (Figure 3).

Macrophage infiltration was detected both within the tumor parenchyma (intra-tumoral localization) and in the adjacent stromal tissue (peritumoral localization). The peritumoral zone often showed a higher density of CD163^+^ cells, while CD68^+^ macrophages were more evenly distributed across both compartments.

In contrast, in the adjacent non-tumorous skin, only isolated CD68^+^ macrophages were observed in the dermis, mostly around blood vessels or hair follicles. CD163^+^ macrophages in these regions were rare or absent.

### 3.4. Quantitative Analysis of Cd68^+^ and Cd163^+^ Macrophage Infiltration According to Breslow Thickness

**Figure 4:**
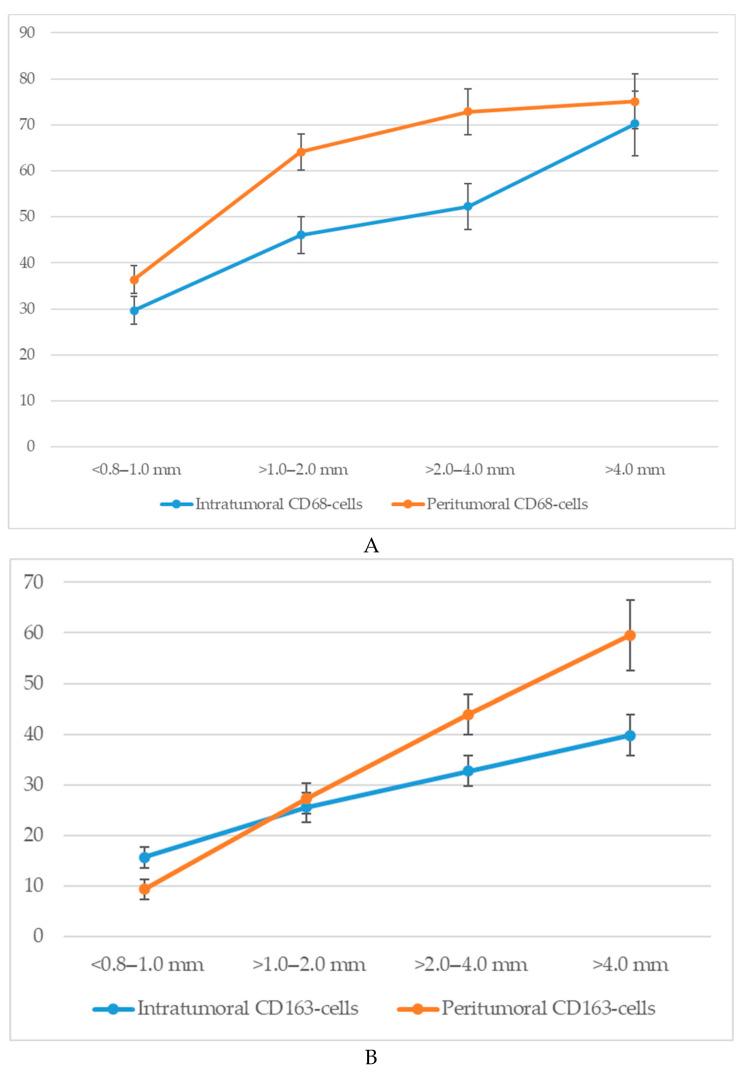
The number of macrophages depending on the thickness according to Breslow (*p* < 0.05): (**A**)—CD68-cells; (**B**)—CD163-cells.

An analysis of macrophage infiltration in cutaneous melanoma based on tumor thickness according to the Breslow scale revealed a clear trend toward an increase in both intratumoral and peritumoral numbers of CD68^+^ and CD163^+^ macrophages with tumor progression. The mean quantitative values of macrophages in the corresponding zones are presented in Figure 4.

Specifically, when tumor thickness was less than 1.0 mm (<0.8–1.0 mm), the average number of intratumoral CD68^+^ macrophages was 29.7 ± 4.3 cells, whereas at a thickness greater than 4.0 mm, this value increased to 70.3 ± 6.4 cells. In the peritumoral zone, the respective values were 36.4 ± 3.6 and 75.1 ± 7.2 cells.

A similar dynamic was observed for CD163^+^ macrophages. In the intratumoral area, tumors with a thickness of <1.0 mm showed a mean of 15.6 ± 2.8 CD163^+^ cells, which increased to 39.8 ± 4.6 cells in tumors >4.0 mm thick. The peritumoral compartment showed an even more pronounced increase from 9.3 ± 2.1 to 59.6 ± 7.3 cells.

Thus, with increasing Breslow thickness, there is a consistent rise in macrophage infiltration both within the tumor mass and in the surrounding stroma. CD68^+^ macrophages show higher absolute counts, whereas CD163^+^ macrophages—particularly in the peritumoral region—exhibit a more pronounced relative increase.

### 3.5. Quantitative Analysis of Cd68^+^ and Cd163^+^ Macrophage Infiltration According to Pt Stage

**Figure 5:**
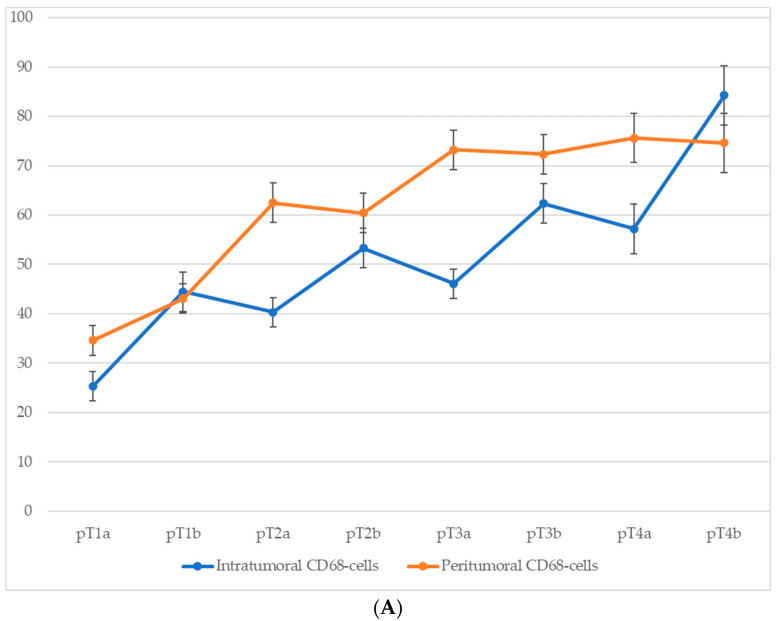

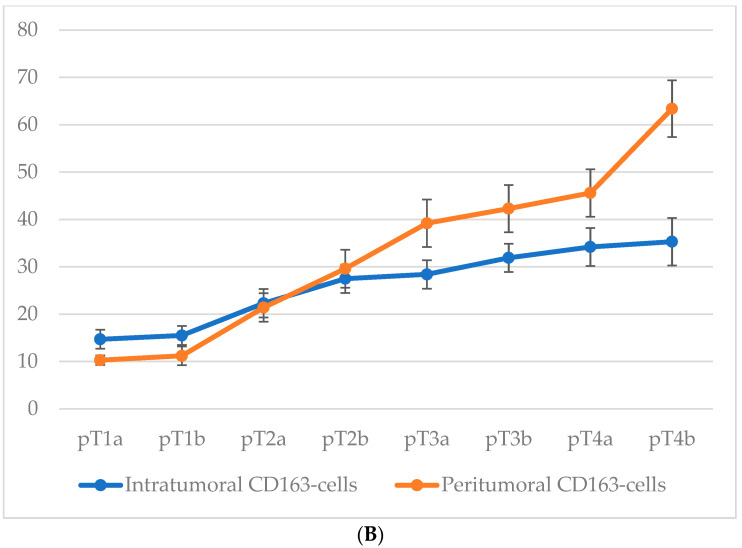
The number of macrophages depending on the pT stage (*p* < 0.05): (**A**)—CD68-cells; (**B**)—CD163-cells.

Comparison of macrophage infiltration levels across different pT stages of cutaneous melanoma revealed a consistent upward trend in both intratumoral and peritumoral CD68^+^ macrophage counts as the tumor stage advanced (Figure 5). This increase was particularly pronounced at stages associated with ulceration (pT1b, pT2b, pT3b, and pT4b), suggesting that intratumoral macrophage accumulation is closely linked with more aggressive tumor behavior. Although peritumoral macrophage density also increased progressively with pT stage, this parameter appeared to be less sensitive to the presence of ulceration, further emphasizing the potential prognostic relevance of intratumoral macrophage infiltration.

For CD68^+^ macrophages, the average number of cells in the intratumoral area increased from 25.3 ± 3.1 at stage pT1a to 59.8 ± 6.3 at stage pT4b, with a particularly steep rise between stages pT2a and pT4b. In the peritumoral zone, the count rose from 30.7 ± 3.2 at pT1a to 68.2 ± 6.4 at pT4b.

A similar trend was observed for CD163^+^ macrophages. In the intratumoral compartment, the average number of CD163^+^ cells increased from 14.7 ± 2.6 at stage pT1a to 35.3 ± 4.8 at stage pT4b, with a particularly steep rise occurring between stages pT2a and pT4b. Peritumoral infiltration of CD163^+^ cells showed an even more pronounced dynamic, ranging from 10.3 ± 1.6 cells at pT1a to 63.4 ± 6.1 at pT4b.

These data highlight not only the correlation between macrophage infiltration and tumor progression but also the more robust relative increase in CD163^+^ cells in the peritumoral area.

### 3.6. Quantitative Analysis of Cd68^+^ and Cd163^+^ Macrophage Infiltration According to Ajcc Stage

**Figure 6:**
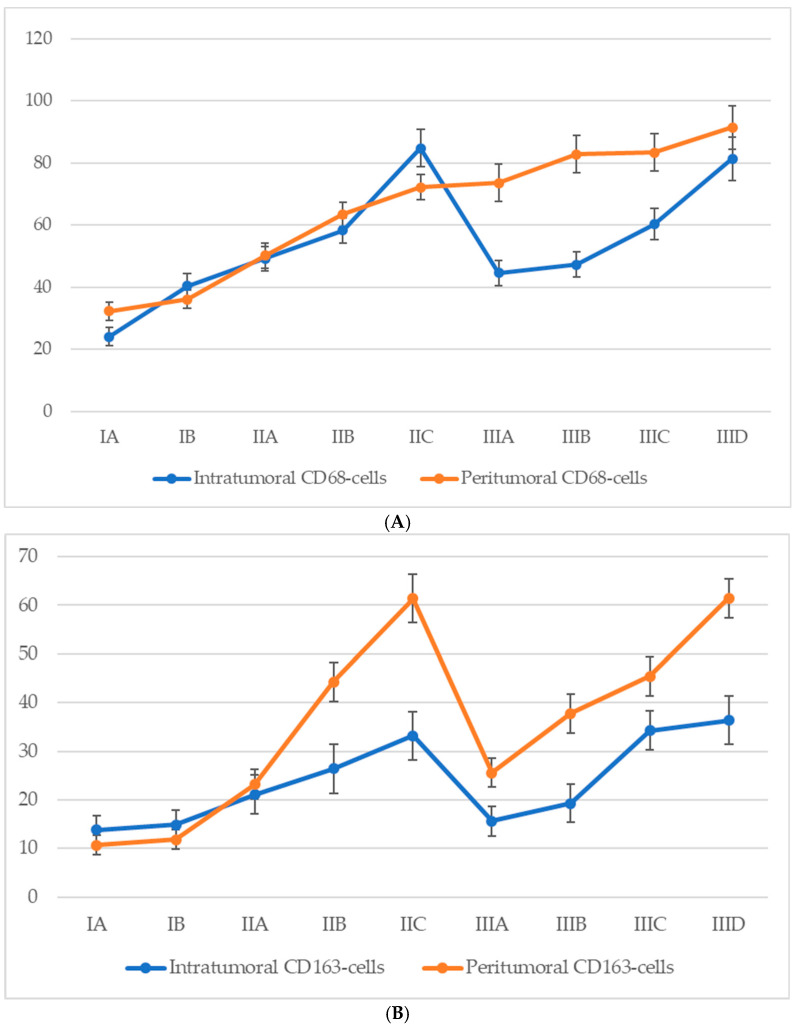
The number of macrophages depending on the AJCC stage (*p* < 0.05): (**A**)—CD68-cells; (**B**)—CD163-cells.

Analysis of macrophage infiltration in relation to the American Joint Committee on Cancer (AJCC, 8th edition) staging system revealed a general trend of increasing CD68^+^ macrophage density in both intratumoral and peritumoral compartments as melanoma progressed (Figure 6). In early-stage disease (IA–IB), macrophage infiltration was moderate, with a predominance of peritumoral accumulation. During stage II, a marked increase was observed in both compartments, with intratumoral CD68^+^ macrophage counts peaking at stage IIC. Interestingly, a decline in intratumoral CD68^+^ macrophage numbers was observed at the transition to stage III (notably at IIIA), despite a continued increase in peritumoral macrophage density. At later substages of stage III (IIIC–IIID), macrophage numbers in both zones began to rise again, approaching or even surpassing the earlier peak values noted at stage IIC.

For CD68^+^ macrophages, the average count in the intratumoral compartment increased from 21.6 ± 3.1 cells at stage IA to 56.4 ± 6.8 cells at stage IIID, with a peak at stage IIC (60.3 ± 6.2 cells). However, a slight decline was observed at stage IIIA (45.7 ± 6.3 cells), despite a continued increase in peritumoral density. In the peritumoral zone, CD68^+^ macrophages showed a steady rise from 28.2 ± 3.2 cells at stage IA to 71.3 ± 7.4 cells at stage IIID, with the most significant increase between stages IIB and IIC (from 35.4 ± 4.2 to 61.7 ± 6.3 cells).

A comparable yet distinct pattern was seen for CD163^+^ macrophages. In the intratumoral region, CD163^+^ cells steadily increased from 13.8 ± 3.2 cells at stage IA to a maximum of 36.4 ± 4.6 cells at stage IIID. However, a mild decline was recorded at stage IIIA (15.6 ± 2.5 cells) before rising again at stages IIIC and IIID. In the peritumoral region, the CD163^+^ cell count demonstrated a more pronounced and sustained increase, from 10.7 ± 2.4 cells at stage IA to 61.5 ± 4.2 cells at stage IIID, with a particularly steep rise during stage II (from 12.4 ± 2.7 to 52.8 ± 4.8 cells), peaking at stage IIC (61.4 ± 4.3 cells), and maintaining consistently high values through advanced stage III.

These findings indicate a progressive enrichment of both CD68^+^ and CD163^+^ macrophages with increasing AJCC stage, with CD163^+^ cells—especially in the peritumoral region—showing a strong and continuous association with advanced disease.

## 4. Discussion

This study aimed to quantitatively assess CD68^+^ and CD163^+^ macrophages in the intratumoral and peritumoral stroma of cutaneous melanoma at different stages of the disease. The results confirmed a consistent trend toward increased macrophage infiltration in both compartments as the tumor progressed. This was clearly observed in the analyses stratified by Breslow thickness, pT stage, and AJCC staging.

One of the key findings was the more pronounced increase in the number of intra-tumoral macrophages at stages characterized by ulceration (pT stages with the “b” index). This may suggest a potential association between melanoma ulceration and the activation of local inflammatory responses, accompanied by enhanced macrophage migration into the tumor mass [16]. At the same time, peritumoral macrophages showed a more gradual but steady increase, less directly associated with ulceration. This may be due to the fact that peritumoral macrophages are located outside the main tumor mass, within the surrounding stromal tissue, where their recruitment is likely driven by chronic inflammation, tumor invasion, and stromal remodeling, rather than by acute damage related to ulceration [17].

An interesting feature was the decrease in intratumoral macrophage counts during the transition from stage II to stage III according to the AJCC classification. This decline may reflect the fact that in stage III melanoma, despite the presence of regional metastases, the depth of dermal invasion is often less than that seen in stages IIB–IIC. Nonetheless, peritumoral macrophage infiltration continued to increase, potentially reflecting a compensatory or protective response aimed at limiting lymphatic dissemination [18]. This pattern highlights the importance of spatial analysis of tumor-associated inflammation in the assessment of prognostic factors and mechanisms of melanoma progression.

Our data are consistent with several previous studies that have demonstrated a positive correlation between macrophage density and tumor thickness, level of invasion, and stage of disease [19,20]. Moreover, our emphasis on differentiating macrophages by their spatial localization provided a more nuanced understanding of the inflammatory component of the tumor microenvironment.

Analysis of CD163^+^ macrophage infiltration revealed similar trends. Both intra-tumoral and peritumoral CD163^+^ macrophage counts increased with advancing tumor stage, with the most pronounced relative increase observed in the peritumoral compartment. This suggests that the accumulation of M2-polarized macrophages may be particularly relevant in the tumor periphery, potentially contributing to immune evasion and promoting a microenvironment favorable to tumor invasion and metastasis [21].

Given that CD163 expression is considered a reliable marker of M2-polarized macrophages—associated with immunosuppressive and tumor-promoting activity—further investigation into the role of these cells in melanoma progression is of significant scientific and clinical interest [22]. In particular, the concurrent use of additional polarization markers, such as CD206, may enable more accurate identification of TAM (tumor-associated macrophage) subtypes and help clarify their contribution to microenvironment remodeling, suppression of anti-tumor immune responses, and the development of prognostic models [23].

Several recent studies further highlight the complex role of tumor-associated macrophages (TAMs) in melanoma. Salmi S. et al. demonstrated that high CD68^+^ macrophage density in tumor nests and low stromal CD163^+^ proportions were associated with recurrence and poor survival, emphasizing the importance of TAM localization [19]. Tremble et al. reported a close link between TAM infiltration and angiogenic signaling, particularly via *VEGF*, suggesting that TAMs promote vascular remodeling and tumor progression [24]. Similarly, Lee et al. showed that CD163, but not CD68, correlated with VEGF and COX−2 expression, and that high CD163 expression was independently associated with deeper Breslow thickness, advanced stage, and worse survival [25]. Finally, De Logu et al. identified a mechanistic connection between CD163^+^ TAM infiltration, oxidative stress, and TRPA1 activation in melanoma cells, showing that TAM-derived ROS can amplify oxidative stress signaling and thereby enhance tumor progression [26]. Together, these findings support our results and underline the need for more detailed analyses of TAM subsets, their microanatomical distribution, and functional interactions with other components of the tumor microenvironment.

Thus, the macrophage infiltration dynamics identified at different stages of cutaneous melanoma progression confirm the importance of this cell population in the pathogenesis of the disease. These results may serve as a foundation for future studies aimed at developing immunotherapeutic approaches targeting the macrophage compartment of the tumor microenvironment.

This study has several strengths, including a relatively large and well-characterized cohort of melanoma patients, the use of standardized immunohistochemical methods, and a quantitative assessment of macrophage infiltration in both intratumoral and peritumoral compartments. The spatially resolved approach allowed us to distinguish different patterns of CD68^+^ and CD163^+^ macrophage distribution across Breslow thickness, pT stage, and AJCC stage. Importantly, our results also provide an opportunity for a comparative analysis of the relative abundance of macrophages with M1- and M2-like phenotypes at different stages of melanoma progression, thereby contributing to a better understanding of their potential roles in tumor development.

However, certain limitations should be acknowledged. The retrospective design and single-center nature of the study may limit the generalizability of our findings. Our study was based on the use of CD68 and CD163 as markers of macrophages, which are widely applied in clinical and research practice. Nevertheless, future studies would benefit from the application of more advanced histological and imaging techniques, such as fluorescent or multiplex immunohistochemistry, with additional markers including pSTAT1, INOS, CD80, and CD86, in order to obtain a more detailed characterization of macrophage subpopulations in melanoma.

## 5. Conclusions

The results of this study demonstrate a clear increase in both CD68^+^ and CD163^+^ macrophage infiltration in cutaneous melanoma tissues as tumor thickness and stage progress. Notably, CD163^+^ macrophages showed a pronounced relative increase, especially in the peritumoral stroma, suggesting their significant role in tumor microenvironment remodeling. The higher intratumoral macrophage density observed in ulcerated melanoma stages highlights a potential link between local inflammatory response and tumor aggressiveness.

Further investigations incorporating macrophage polarization markers and spatial profiling techniques are necessary to better understand the functional roles of distinct macrophage subsets in melanoma progression. Additionally, longitudinal studies correlating macrophage infiltration with patient outcomes will be essential for validating these immune cells as prognostic markers and potential targets for immunotherapeutic interventions.

## Figures and Tables

**Table 1:** Clinicopathological Characteristics of the Patients.

Characteristic	Rate
Age	29–88 years
Average age	64.2 ± 6.8 years
Gender	
Male	55
Female	71
Histologic type	
Superficial spreading melanoma	46
Nodular melanoma	69
Desmoplastic melanoma	11
Breslow thickness	
<0.8–1.0 mm	32
>1.0–2.0 mm	28
>2.0–4.0 mm	35
>4.0 mm	31
Clark level of invasion	
I	16
II	25
III	24
IV	49
V	12
Localization of primary melanoma	
Head and neck	19
Trunk	31
Upper extremities	29
Lower extremities	47
pT	
pT1a	23
pT1b	9
pT2a	20
pT2b	8
pT3a	21
pT3b	14
pT4a	17
pT4b	14
IA	23
IB	10
IIA	12
IIB	14
IIC	14
IIIA	17
IIIB	17
IIIC	17
IIID	2

## Data Availability

The study did not generate publicly available archival data.

## References

[1] Arnold M., Singh D., Laversanne M., Vignat J., Vaccarella S., Meheus F., Cust A.E., de Vries E., Whiteman D.C., Bray F. (2022). Global Burden of Cutaneous Melanoma in 2020 and Projections to 2040. JAMA Dermatol..

[2] Tímár J., Ladányi A. (2022). Molecular Pathology of Skin Melanoma: Epidemiology, Differential Diagnostics, Prognosis and Therapy Prediction. Int. J. Mol. Sci..

[3] Federico S., Fortarezza F., Ingravallo G., Cazzato G. (2025). Epidemiology of Skin Cancer in 2024.

[4] Bray F., Laversanne M., Sung H., Ferlay J., Siegel R.L., Soerjomataram I., Jemal A. (2024). Global cancer statistics 2022: GLOBOCAN estimates of incidence and mortality worldwide for 36 cancers in 185 countries. CA Cancer J. Clin..

[5] Marzagalli M., Ebelt N.D., Manuel E.R. (2019). Unraveling the crosstalk between melanoma and immune cells in the tumor microenvironment. Semin. Cancer Biol..

[6] Kharouf N., Flanagan T.W., Hassan S.Y., Shalaby H., Khabaz M., Hassan S.-L., Megahed M., Haikel Y., Santourlidis S., Hassan M. (2023). Tumor Microenvironment as a Therapeutic Target in Melanoma Treatment. Cancers.

[7] Mihulecea C.R., Ceaușu R.A., Gaje N.P., Rotaru M., Raica M. (2024). Review: The tumor microenvironment of melanoma. Med. Evol..

[8] Wądzyńska J., Simiczyjew A., Pietraszek-Gremplewicz K., Kot M., Ziętek M., Matkowski R., Nowak D. (2023). The impact of cellular elements of TME on melanoma biology and its sensitivity to EGFR and MET targeted therapy. Mol. Cell Res..

[9] Haroon S., Rashid K., Elmahdy H., Zia S., Malik U.A., Irfan M., Hashmi A.A. (2023). Clinicopathological Profile of a Cohort of Patients with Malignant Melanoma in the United Kingdom. Cureus.

[10] Lima-Pérez M., Mejía J., Mateus G. (2023). Caracterización histológica del melanoma cutáneo en reportes de patología en la ciudad de Cali, 2016–2021. Rev. Colomb. Cir..

[11] Pampena R., Lai M., Lombardi M., Mirra M., Raucci M., Lallas A., Apalla Z., Argenziano G., Pellacani G., Longo C. (2020). Clinical and Dermoscopic Features Associated with Difficult-to-Recognize Variants of Cutaneous Melanoma: A Systematic Review. JAMA Dermatol..

[12] Gutiérrez-Castañeda L.D., Nova J.A., Tovar-Parra J.D. (2020). Frequency of mutations in BRAF, NRAS, and KIT in different populations and histological subtypes of melanoma: A systemic review. Melanoma Res..

[13] Kaneko M., Saito R., Nishikawa A., Ito A. (2025). All-In-One Magnetic Nanoparticles for Thermo-Immunotherapy of Malignant Melanoma. Adv. Healthc. Mater..

[14] Asai Y., Yanagawa N., Osakabe M., Yamada N., Sugimoto R., Sato A., Ito K., Koike Y., Tanji T., Sakuraba M. (2024). The clinicopathological impact of tumor-associated macrophages in patients with cutaneous malignant melanoma. J. Surg. Oncol..

[15] Bankhead P., Loughrey M.B., Fernández J.A., Dombrowski Y., McArt D.G., Dunne P.D., McQuaid S., Gray R.T., Murray L.J., Coleman H.G. (2017). QuPath: Open source software for digital pathology image analysis. Sci. Rep..

[16] Aitcheson S.M., Frentiu F.D., Hurn S.E., Edwards K., Murray R.Z. (2021). Skin Wound Healing: Normal Macrophage Function and Macrophage Dysfunction in Diabetic Wounds. Molecules.

[17] Ramakrishnan G., Miskolci V., Hunter M.V., Giese M.A., Münch D., Hou Y., Eliceiri K.W., Lasarev M.R., White R.M., Huttenlocher A. (2024). Real-time imaging reveals a role for macrophage protrusive motility in melanoma invasion. J. Cell Biol..

[18] Hu D., Liu Z., Chen S., Huang Y., Zeng W., Wei W., Zhang C., Zhou L., Chen D., Wu Y. (2022). Assessment of the Novel, Practical, and Prognosis-Relevant TNM Staging System for Stage I–III Cutaneous Melanoma. Front. Oncol..

[19] Salmi S., Siiskonen H., Sironen R., Tyynelä-Korhonen K., Hirschovits-Gerz B., Valkonen M., Auvinen P., Pasonen-Seppänen S. (2019). The number and localization of CD68^+^ and CD163^+^ macrophages in different stages of cutaneous melanoma. Melanoma Res..

[20] Li Z., Zhang X., Jin Q., Zhang Q., Yue Q., Fujimoto M., Jin G. (2023). Development of a Macrophage-Related Risk Model for Metastatic Melanoma. Int. J. Mol. Sci..

[21] Boutilier A.J., Elsawa S.F. (2021). Macrophage Polarization States in the Tumor Microenvironment. Int. J. Mol. Sci..

[22] Zhao R., Zhang X., Geng Y., Lu D., Wang Y., Xie H., Zhang X., Xu S., Cao Y. (2025). SPRY1 regulates macrophage M1 polarization in skin aging and melanoma prognosis. Transl. Oncol..

[23] Scortegagna M., Murad R., Bina P., Feng Y., Porritt R.A., Terskikh A.V., Tian X., Adams P.D., Vuori K., Ronai Z.A. (2025). Age-Associated Modulation of TREM1/2-Expressing Macrophages Promotes Melanoma Progression and Metastasis. Cancer Res..

[24] Tremble L.F., McCabe M., Walker S.P., McCarthy S., Tynan R.F., Beecher S., Werner R., Clover A.J.P., Power X.D.G., Forde P.F. (2020). Differential association of CD68^+^ and CD163^+^ macrophages with macrophage enzymes, whole tumour gene expression and overall survival in advanced melanoma. Br. J. Cancer.

[25] Lee W.J., Lee M.H., Kim H.T., Won C.H., Lee M.W., Choi J.H., Chang S.E. (2019). Prognostic significance of CD163 expression and its correlation with cyclooxygenase-2 and vascular endothelial growth factor expression in cutaneous melanoma. Melanoma Res..

[26] De Logu F., Souza Monteiro de Araujo D., Ugolini F., Iannone L.F., Vannucchi M., Portelli F., Landini L., Titiz M., De Giorgi V., Geppetti P. (2021). The TRPA1 Channel Amplifies the Oxidative Stress Signal in Melanoma. Cells.

